# Neutrophil and Eosinophil Responses Remain Abnormal for Several Months in Primary Care Patients With COVID-19 Disease

**DOI:** 10.3389/falgy.2022.942699

**Published:** 2022-07-27

**Authors:** B. N. Jukema, K. Smit, M. T. E. Hopman, C. C. W. G. Bongers, T. C. Pelgrim, M. H. Rijk, T. N. Platteel, R. P. Venekamp, D. L. M. Zwart, F. H. Rutten, L. Koenderman

**Affiliations:** ^1^Department of Respiratory Medicine, University Medical Center Utrecht, Utrecht University, Utrecht, Netherlands; ^2^Center for Translational Immunology, University Medical Center Utrecht, Utrecht University, Utrecht, Netherlands; ^3^Department of General Practice, Julius Center for Health Sciences and Primary Care, University Medical Center Utrecht, Utrecht University, Utrecht, Netherlands; ^4^Department of Physiology, Radboud Institute for Health Sciences, Radboud University Medical Center, Nijmegen, Netherlands

**Keywords:** SARS-CoV-2, primary care, granulocytes, flow cytometry, activation, long COVID

## Abstract

**Introduction:**

Neutrophil and eosinophil activation and its relation to disease severity has been understudied in primary care patients with COVID-19. In this study, we investigated whether the neutrophil and eosinophil compartment were affected in primary care patients with COVID-19.

**Methods:**

COVID-19 patients, aged ≥ 40 years with cardiovascular comorbidity presenting to the general practitioner with substantial symptoms, partaking in the COVIDSat@Home study between January and April 2021, were included. Blood was drawn during and 3 to 6 months after active COVID-19 disease and analyzed by automated flow cytometry, before and after stimulation with a formyl-peptide (fNLF). Mature neutrophil and eosinophil markers at both time points were compared to healthy controls. A questionnaire was conducted on disease symptoms during and 3 to 6 months after COVID-19 disease.

**Results:**

The blood of 18 COVID-19 patients and 34 healthy controls was analyzed. During active COVID-19 disease, neutrophils showed reduced CD10 (*p* = 0.0360), increased CD11b (*p* = 0.0002) and decreased CD62L expression (*p* < 0.0001) compared to healthy controls. During active COVID-19 disease, fNLF stimulated neutrophils showed decreased CD10 levels (*p* < 0.0001). Three to six months after COVID-19 disease, unstimulated neutrophils showed lowered CD62L expression (*p* = 0.0003) and stimulated neutrophils had decreased CD10 expression (*p* = 0.0483) compared to healthy controls. Both (un)stimulated CD10 levels increased 3 to 6 months after active disease (*p* = 0.0120 and *p* < 0.0001, respectively) compared to during active disease. Eosinophil blood counts were reduced during active COVID-19 disease and increased 3 to 6 months after infection (*p* < 0.0001). During active COVID-19, eosinophils showed increased unstimulated CD11b (*p* = 0.0139) and decreased (un)stimulated CD62L expression (*p* = 0.0036 and *p* = 0.0156, respectively) compared to healthy controls. Three to six months after COVID-19 disease, (un)stimulated eosinophil CD62L expression was decreased (*p* = 0.0148 and *p* = 0.0063, respectively) and the percentage of CD11b^bright^ cells was increased (*p* = 0.0083 and *p* = 0.0307, respectively) compared to healthy controls.

**Conclusion:**

Automated flow cytometry analysis reveals specific mature neutrophil and eosinophil activation patterns in primary care patients with COVID-19 disease, during and 3 to 6 months after active disease. This suggests that the neutrophil and eosinophil compartment are long-term affected by COVID-19 in primary care patients. This indicates that these compartments may be involved in the pathogenesis of long COVID.

## Introduction

The 2019 coronavirus disease (COVID-19) is associated with a wide spectrum of symptoms, ranging from asymptomatic to critical disease (1, 2). After the initial infection and active COVID-19 disease, patients may have persistent (residual) symptoms for weeks, months or even years. These symptoms are collectively referred to as long COVID (3).

The potential role of neutrophils in COVID-19 pathogenesis and the relation to disease severity has been studied thoroughly in hospitalized patients (4–7). Interestingly, a maturation dissociation in neutrophils, characterized by a low expression of CD10, was found amongst COVID-19 patients who presented at the emergency department (4). Moreover, this maturation dissociation was present (within days) after initial symptom onset.

Previous research showed that, for eosinophils, eosinopenia has been associated with COVID-19 (8). In depth analysis in COVID-19 patients presenting to the emergency department revealed altered responsiveness of eosinophils to *ex vivo* activation, also within days after symptom onset (9). Collectively, these findings suggests a potential role for granulocytes in the pathogenesis of COVID-19.

Nevertheless, the role of neutrophils and eosinophils and its relation to disease severity is poorly studied in patients presenting to primary care with COVID-19. This is a major omission since the majority of COVID-19 patients are managed in primary care. This study therefore investigated whether the neutrophil and eosinophil compartment were affected in patients presenting to primary care with cardiovascular comorbidity and COVID-19, both during and 3 to 6 months after active disease. Furthermore, we investigated whether aberrant responsiveness of granulocytes to formyl-peptides were associated with prolonged symptoms 3 to 6 months after active disease.

## Materials and Methods

### Study Design and Population

The study population consisted of participants of CovidSat@Home, a primary care-based, open, randomized controlled pilot trial aimed to assess the feasibility of a trial of home monitoring of COVID-19 patients by pulse oximetry (10). All patients that were included in the trial were aged ≥ 40 years with cardiovascular comorbidity who presented to the general practitioner with COVID-19 symptoms defined as at least 3 days (I) a core temperature ≥37.5°C, (II) a feeling of shortness of breath in rest (or with minimal exercise) and/or (III) sudden exhaustion, and with the necessity of close-follow up according to the general practitioner. All participants had a positive SARS-CoV-2 reverse transcription polymerase chain reaction test between 19 January 2021 and 28 April 2021.

**Figure 1:**
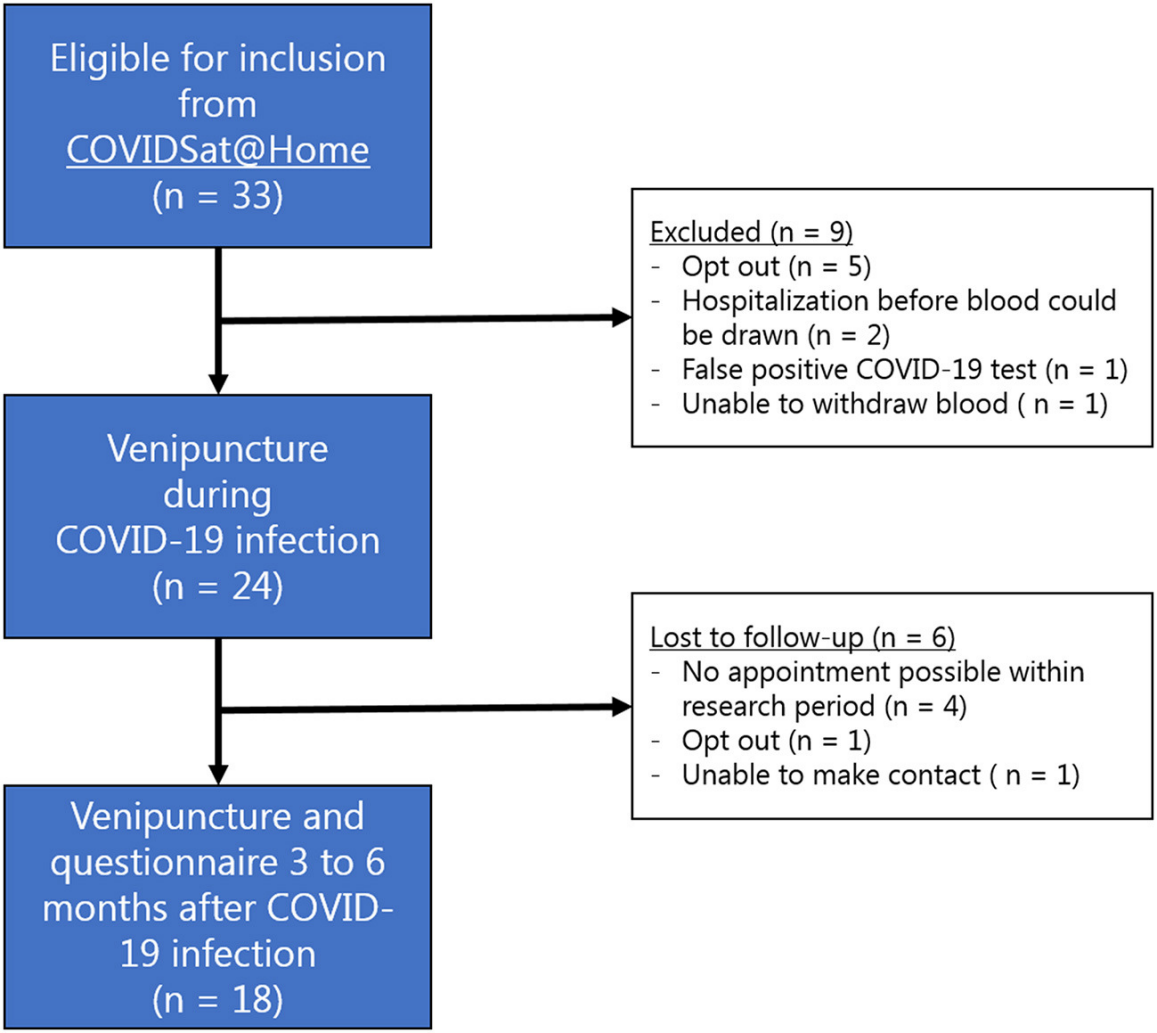
Flowchart depicting the inclusion of patients for this study.

After written informed consent was obtained for this bolt-on study, the first blood sample was drawn at home during the initial phase of COVID-19 disease by a registered medical doctor from the study team. This time point was defined as “during active disease.” Three to six months after active COVID-19 disease, the patients were asked for renewed consent to have a second blood sample drawn at home. This time point was defined as “after active disease.” At this moment, a standardized questionnaire on presence of COVID-19 related symptoms during and 3 to 6 months after active disease was completed (see Appendix). Patients in which no second blood sample was drawn were excluded and labeled as lost to follow-up (Figure 1). Collected blood tubes were immediately transported to the University Medical Center Utrecht and analyzed by the automated AQUIOS CL^®^ “Load & Go” flow cytometer (Beckman Coulter, Indianapolis, IN, USA), which is point of care located at the emergency department.

### Healthy Controls

Blood from healthy controls was obtained from healthy individuals participating in the Nijmegen Exercise Study 2021. In this study the effect of protein supplementation on muscle force and soreness was investigated during several days before and after hiking for 20–30 km in healthy individuals. The blood of the healthy controls was drawn before exercise and analyzed by AQUIOS CL identically as the blood of the COVID-19 patient cohort in this study.

Neutrophils get easily activated by *ex vivo* circumstances and manipulations in a time dependent manner (11). Therefore, the time of venipuncture till analysis was registered for both the patients and healthy controls. The healthy control cohort was matched to the patients based on this time till analysis range (25–90 min). To rule out time till analysis-bias, healthy control samples that were analyzed beyond this timeframe were excluded. Eventually, 34 healthy controls could be included in the study.

### Automated Flow Cytometry Analysis

The AQUIOS CL automatically prepares and performs flow cytometry analysis of blood samples (11). In short, a 4 ml sodium heparin Vacuette^®^ blood collecting tube (Greiner Bio-One, Kremsmünster, Austria) was placed into a cassette into the device. The device mixed and pipetted the blood into a 96-deep well plate and stained the blood for 15 min with a predefined study antibody-mix for granulocytes. To investigate cell reactivity, every sample was analyzed both in the absence and the presence of the activator N-Formyl-norleucyl-leucyl-phenylalanine (fNLF; end concentration 10^−5^ M; BioCat GmbH, Heidelberg, Germany) in the deep well plate. The predefined antibody-mix (all from Beckman Coulter) contained CD16-FITC (clone 3G8), CD11b-PE (clone Bear1), CD62L-ECD (clone DREG56), CD10-PC5 (clone ALB1), CD64-PC7 (clone 22). Next, the red blood cells were lysed by adding 335 μl AQUIOS Lysing Reagent A (a cyanide-free lytic). The lysis was stopped after 30 s by adding 100 μl AQUIOS Lysing Reagent B, followed by aspiration and analysis through the flow cell.

To validate the identification of eosinophils in the study granulocyte panel, the samples of 22 patients were also analyzed with another antibody-mix, specifically for eosinophils. The eosinophil panel contains CD193-FITC (BioLegend, clone 5E8), CD44-PE, CD62L-ECD, CD16-PC5 and CD11b-PC7 (Beckman Coulter, clone A32537, clone IM2713U, clone A07767 and clone A54822, respectively).

### Data Analysis

**Figure 2:**
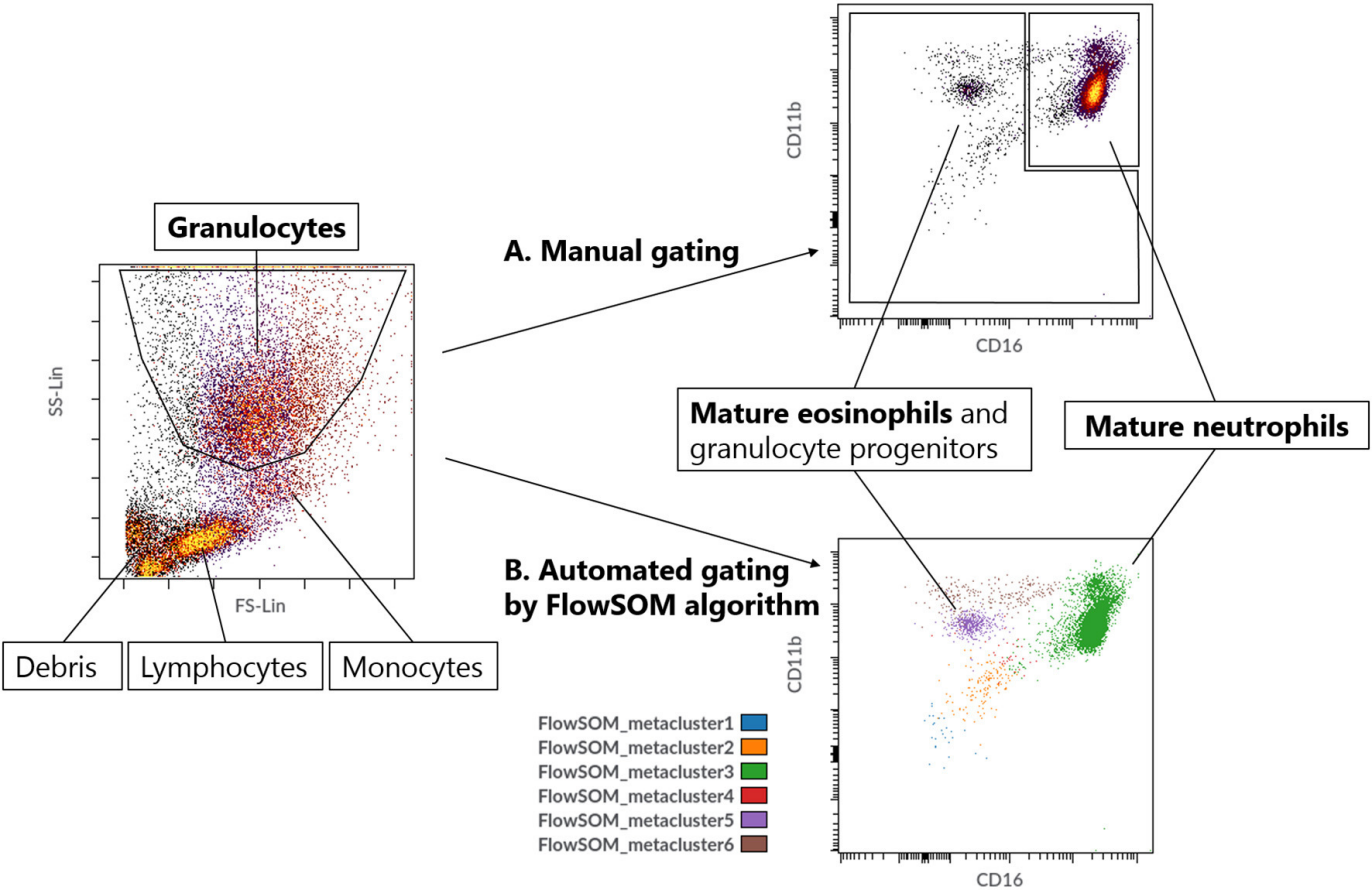
**(A)** Manual gating strategy for mature neutrophils. **(B)** Automated gating strategy for mature neutrophils (metacluster 3) and eosinophils (metacluster 5) by using the FlowSOM algorithm with 64 clusters and 6 metaclusters.

Flow cytometry data files were extracted from the device as FCS 3.1 High Res Listmode Files (.lmd) and imported into Cytobank (www.cytobank.org, a web-based flow cytometry analysis platform; Beckman Coulter, Indianapolis, IN, USA). Granulocytes were gated based on forward scatter (FSC) and sideward scatter (SCC) (Figure 2). Mature neutrophils and eosinophils were automatically gated into 64 clusters and 6 meta-clusters by the FlowSOM algorithm. The mature neutrophil meta-cluster (green) was identified based on containing CD11b^high^ and CD16^high^ cells (12). The eosinophil meta-cluster was identified from the other meta-clusters by combining data from both the study granulocyte panel and eosinophil panel: the purple meta-cluster contains CD11b^high^, CD16^low^ and CD193^high^ cells, identifying them as eosinophils (Supplementary Figure 1). For all further analyses, the green meta-cluster (mature neutrophils) and purple meta-cluster (eosinophils) from the study granulocyte panel were used.

The median fluorescence intensity (MFI) was determined for all aforementioned markers on mature neutrophils and eosinophils. Each sample has two MFI values per marker: one for unstimulated cells (without fNLF) and one for activated cells (with fNLF 10 μM). The percentage of bright eosinophils was determined by using a gate at a predefined MFI (547873 for CD11b and 136968 for CD62L; Supplementary Figure 2).

### Clinical Characteristics

Baseline characteristics of the study population were captured from the patients' electronic health records, and included age, sex category, time till questionnaire after active COVID-19 disease, and hospital admission during follow-up. Also healthy control data was collected with respect to sex, age and relevant disease history over the past year.

### Patient Reported Symptoms

Data on symptoms and complaints (see Appendix) during and 3 to 6 months after active COVID-19 disease were collected. Each symptom was scored for severity on a predefined scale: 1 = not existent, 2 = mild, 3 = moderate, 4 = substantial, 5 = severe. Disease severity was determined by adding up the severity of all symptoms that were present. If symptoms were present prior to the SARS-CoV-2 infection, the change in severity of symptoms was scored.

### Statistical Analysis

Categorical variables are presented as frequencies and percentages. Continuous variables are depicted as mean and standard deviation (SD; parametric distribution) or median and interquartile range (IQR; non-parametric distribution). When comparing two groups, the Chi^2^ test (*n* < 60 samples per cell) or Fisher's exact test (*n* < 5 samples per cell) were used for categorical variables and the Mann-Whitney U Test was used for continuous variables. The Wilcoxon matched-pairs signed rank test was used for paired analyses. GraphPad Prism (version 8.3.0; Graphpad software, Inc., San Diego, CA, USA) was used to visualize the data and calculate MFI statistics. Statistical analysis of baseline characteristics was performed in IBM SPSS statistics (version 26.0.0.1). A *p*-value <0.05 was considered statistically significant.

### Ethical Approval and Trial Registration

This study was reviewed and approved by the Medical Ethics Review Committee Utrecht (20-638/D). The trial has been registered at the Netherlands Trial Register (NL8954), https://www.trialregister.nl/trial/8954.

## Results

### Study Population and Healthy Controls

Between 19 January 2021 and 28 April 2021, a total of 24 of 33 (73%) eligible patients consented for the first venipuncture (Figure 1). From six patients no second blood sample was drawn, leaving 18 patients suitable for inclusion in the analysis (Figure 2). The mean age of these patients was 62 years (SD 10, Table 1). Five patients (28%) were female. The healthy control cohort consisted of 34 participants. The mean age of the healthy controls was 70 years (SD 6) of which four (12%) were female. Age and sex were not significantly different. None of the healthy controls reported a history of COVID-19 disease.

**Table 1:** Baseline characteristics of study population and healthy controls.

**Characteristics**	**Study population (*n* = 18)**	**Healthy controls (*n* = 34)**	***P*-value**
Mean age in years (SD)	62 (10)	70 (6)	0.111
Female	5 (28%)	4 (12%)	0.240
Hospital admission	1 (6%)	-	-
Reported history of COVID-19 disease	-	0 (0%)	-
Median time till second blood sample draw in days (IQR)	171 (122–213)	-	-
Second blood sample drawn within 6 months	13 (72%)	-	-

*Statistical analyses were performed using the Fisher's Exact Test for 2-sided significance*.

### Patient Reported Symptoms During and After Active COVID-19 Disease

Most patients experienced a variety of symptoms during the active COVID-19 disease, such as fever, coughing, shortness of breath, gastro-intestinal symptoms, head- or muscle ache, decreased concentration and fatigue. The median severity of all symptoms was rated as mild (2/5). When assessing individual symptoms, patients reported fever (moderate severity, 3/5) and fatigue (substantial severity, 4/5) as symptoms with an above average symptom burden.

Three to six months after active COVID-19 disease, a third of the patients still experienced one or more COVID-19 related symptom, including coughing, shortness of breath, diarrhea, loss of taste and/or smell. Patients also reported prolonged cognitive symptoms such as decreased concentration, experiencing difficulties with thinking and sleeping problems.

Fatigue was both the most commonly reported symptom during (94.4% of the patients) and 3 to 6 months after (33.3% of the patients with a median symptom burden of moderate severity, 3/5) active COVID-19 disease.

### Neutrophil Surface Markers During and 3 to 6 Months After COVID-19 Disease

#### Mature Neutrophils Are Activated in Primary Care Patients During Active COVID-19 Disease but Are Refractory for Additional Activation by FNLF

Neutrophil data obtained during active COVID-19 disease in primary care patients showed a maturation dissociation when compared to healthy controls.

**Figure 3:**
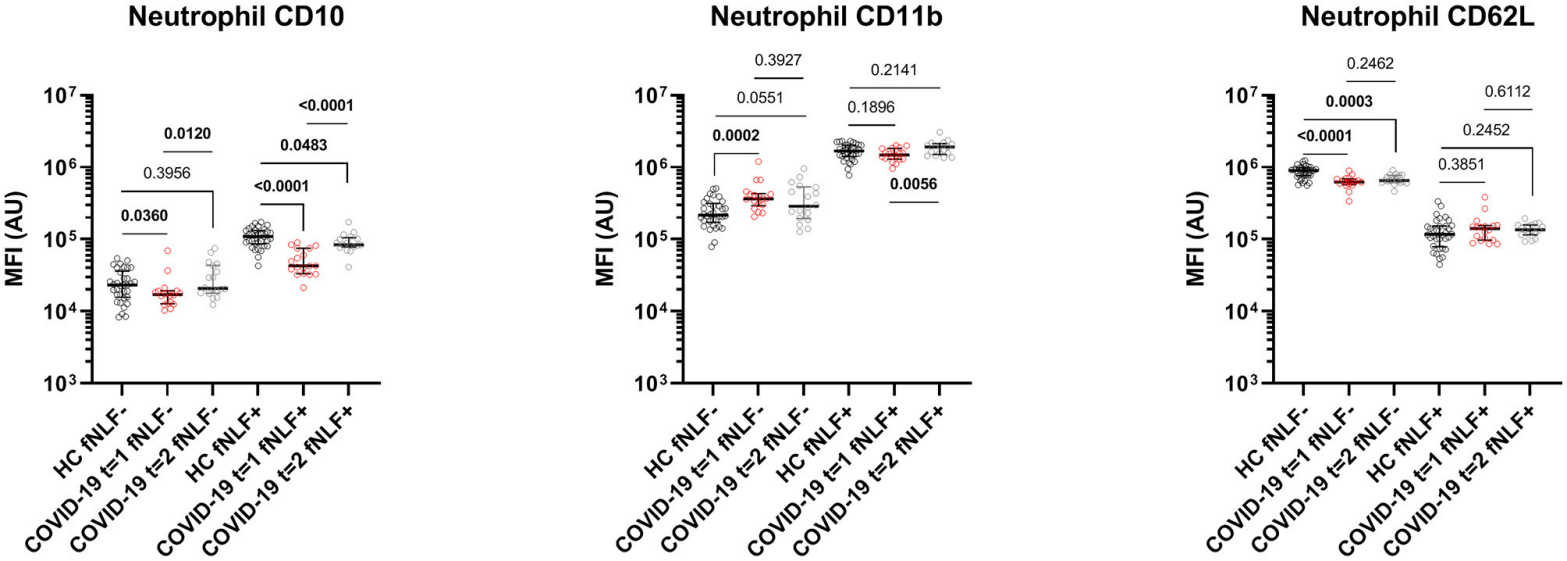
Median fluorescence intensity (MFI) in arbitrary units (AU) for markers on mature neutrophils during (*t* = 1) and 3 to 6 months after (*t* = 2) active COVID-19 disease in primary care patients. Healthy controls (HC) are displayed as reference. fNLF-samples are measured in the absence of fNLF, whereas fNLF+ samples are measured in the presence of the formylpeptide (10 μM). Statistical significance was tested using the Mann-Whitney U Test for continuous variables and the Wilcoxon matched-pairs signed rank test for paired analyses.

The dissociation was characterized by decreased baseline levels of neutrophil CD10 [median MFI, 17 x 10^3^ (IQR 13 x 10^3^-19 x 10^3^) vs. 23 x 10^3^ (IQR 16 x 10^3^-36 x 10^3^), *p* = 0.0360]. Neutrophils from COVID-19 patients also showed increased CD11b levels [median MFI, 37 x10^4^ (IQR 29 x 10^4^-43 x 10^4^) vs. 22 x 10^4^ (IQR 17 x 10^4^-31 x 10^4^), *p* = 0.0002] and decreased CD62L levels [median MFI, 62 x 10^4^ (IQR 57 x 10^4^-69 x 10^4^) vs. 90 x 10^4^ (IQR 76 x 10^4^-98 x 10^4^), *p* < 0.0001, see Figure 3].

**Figure 4:**
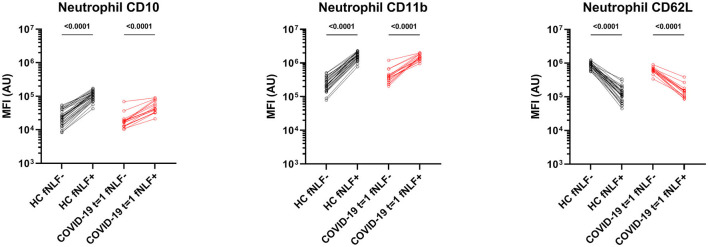
Median fluorescence intensity (MFI) in arbitrary units (AU) for markers on mature neutrophils in healthy controls (HC) and in primary care patients with active COVID-19 disease (*t* = 1). Paired analyses are shown for the samples measured in the absence (fNLF-) and presence (fNLF+) of a formylpeptide (10 μM). Statistical significance was tested using the Wilcoxon matched-pairs signed rank test.

After activating the neutrophils with fNLF, the COVID-19 patients and healthy controls both exhibited increased expression of CD11b and CD10, whilst CD62L expression was decreased (Figure 4). COVID-19 patients showed, compared to healthy controls, decreased CD10 levels after activation [median MFI, 43 x 10^3^ (IQR 33 x 10^3^-75 x 10^3^) vs. 10 x 10^4^ (IQR 86 x 10^3^-13 x 10^4^), *p* < 0.0001), Figure 3]. Expression of CD11b and CD62L after activation with fNLF did not differ significantly.

COVID-19 patients in primary care showed baseline activated mature neutrophils during active COVID-19 disease, as indicated by up- and downregulated surface markers, that were unable to respond to the same extend as healthy controls in response to activation with fNLF.

#### Expression of Mature Neutrophil Activation Markers Is Still Affected 3 to 6 Months After Active COVID-19 Disease

Baseline CD62L expression of unstimulated neutrophils from COVID-19 patients 3 to 6 months after active disease was lowered compared to healthy controls [median MFI, 65 x 10^4^ (IQR 62 x 10^4^- 77 x 10^4^) vs. 90 x 10^4^ (IQR 76 x 10^4^- 98 x 10^4^) *p* = 0.0003, Figure 3]. Baseline CD11b and CD10 expression levels of neutrophils 3 to 6 months after active disease did not differ significantly from the healthy controls.

Due to failed lysis of red blood cells, the data after fNLF activation from one patient was excluded from both neutrophil and eosinophil analyses 3 to 6 months after the active disease. Neutrophils from COVID-19 patients 3 to 6 months after active disease that were stimulated with fNLF exhibited a decreased expression of CD10 [median MFI, 84 x 10^3^ (IQR 78 x 10^3^-11 x 10^4^) vs. 11 x 10^4^ (IQR 89 x 10^3^-13 x 10^4^), *p* = 0.0483, Figure 3], compared to healthy controls. Expression of CD11b and CD62L on fNLF-stimulated neutrophils did not differ from the healthy controls.

Mature neutrophils still showed signs of activation 3 to 6 months after active COVID-19 disease with decreased CD62L expression and mild but statistically significant refractoriness of the cells for activation with formyl peptides as these neutrophils were unable to adequately upregulate CD10 expression after activation with fNLF.

#### Increased Expression of Neutrophil Activation/Differentiation Markers 3 to 6 Months After Active COVID-19 Disease in Comparison With During Active COVID-19 Disease

When compared to neutrophils during active COVID-19 disease, the expression of CD10 on both unstimulated and fNLF-stimulated neutrophils in COVID-19 patients significantly increased three to six months after active COVID-19 disease [unstimulated median MFI, 17 x10^3^ (IQR 1 x 10^3^-19 x 10^3^) vs. 20 x 10^3^ (IQR 18 x 10^3^-44 x 10^3^), *p* = 0.0120; stimulated median MFI 43 x 10^3^ (IQR 33 x 10^3^-75 x 10^3^) vs. 84 x 10^3^ (IQR 78 x 10^3^-10 x 10^4^), *p* < 0.0001, Figure 3]. CD11b expression on fNLF-stimulated neutrophils significantly increased in the period after active disease [median MFI, 15 x 10^5^ (IQR 13 x 10^5^-18 x 10^5^) vs. 19 x 10^5^ (IQR 15 x 10^5^-21 x 10^5^), *p* = 0.0056]. Unstimulated CD11b and both stimulated and unstimulated CD62L levels were similar during and after COVID-19 disease.

### Eosinophil Surface Markers During and 3 to 6 Months After COVID-19 Disease

#### Eosinophil Blood Counts Are Reduced During Active COVID-19 Disease in Primary Care

**Figure 5:**
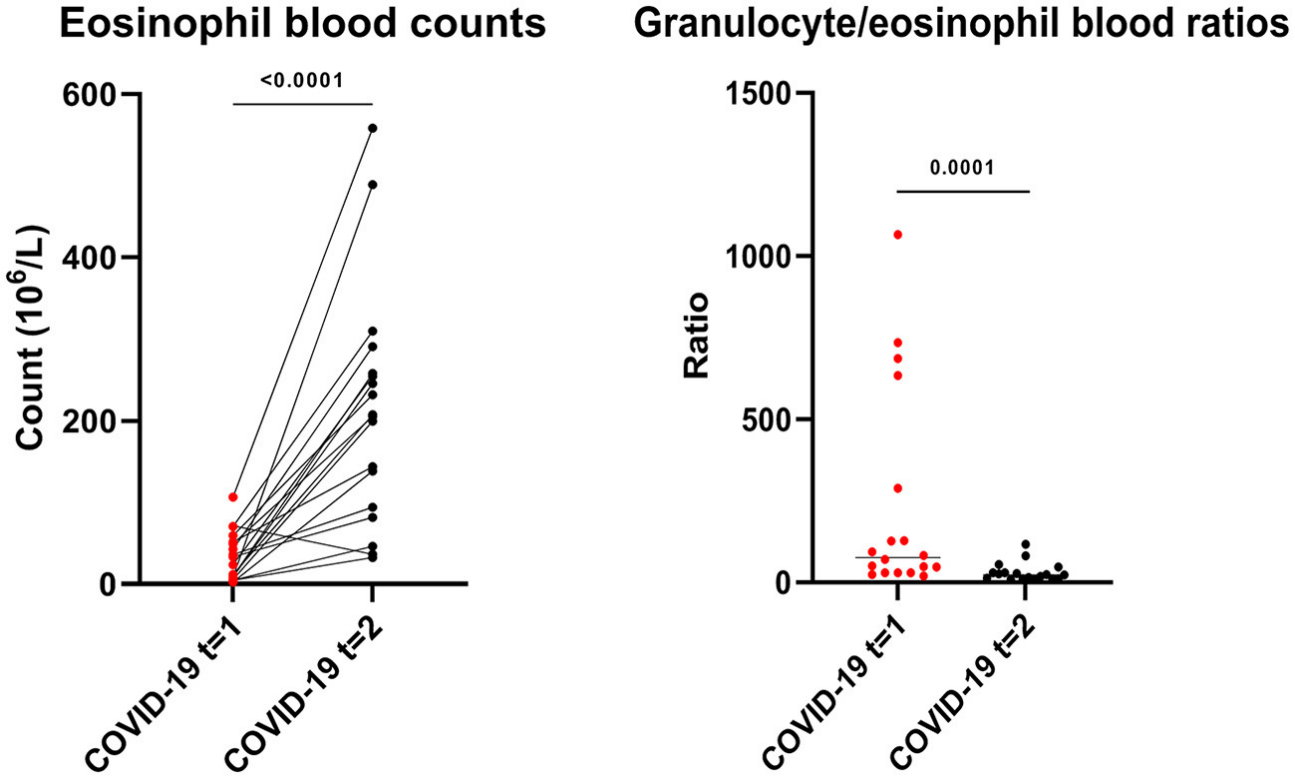
Eosinophil blood counts and granulocyte/eosinophil blood count ratios during (*t* = 1) and 3 to 6 months after (*t* = 2) active COVID-19 disease in primary care patients. Statistical significance was tested using the Wilcoxon matched-pairs signed rank test.

Patients with active COVID-19 disease had lowered eosinophil blood counts, which significantly increased 3 to 6 months after the infection (median blood count, 29 x 10^6^/L (IQR 8 x 10^6^/L-53 x 10^6^/L) vs. 206 x 10^6^/L (IQR 91 x 10^6^L-266 x 10^6^/L), *p* < 0.0001). Granulocyte/eosinophil ratios in peripheral blood significantly increased in the months after COVID-19 when compared to during the active infection (median ratio, 76 (IQR 30–375) vs. 23 (IQR 10–34), *p* = 0.0001, Figure 5). However, eosinophil counts did not fully normalize at this point in time.

#### Eosinophils Show Signs of Activation in Primary Care Patients With Active COVID-19 Disease

**Figure 6:**
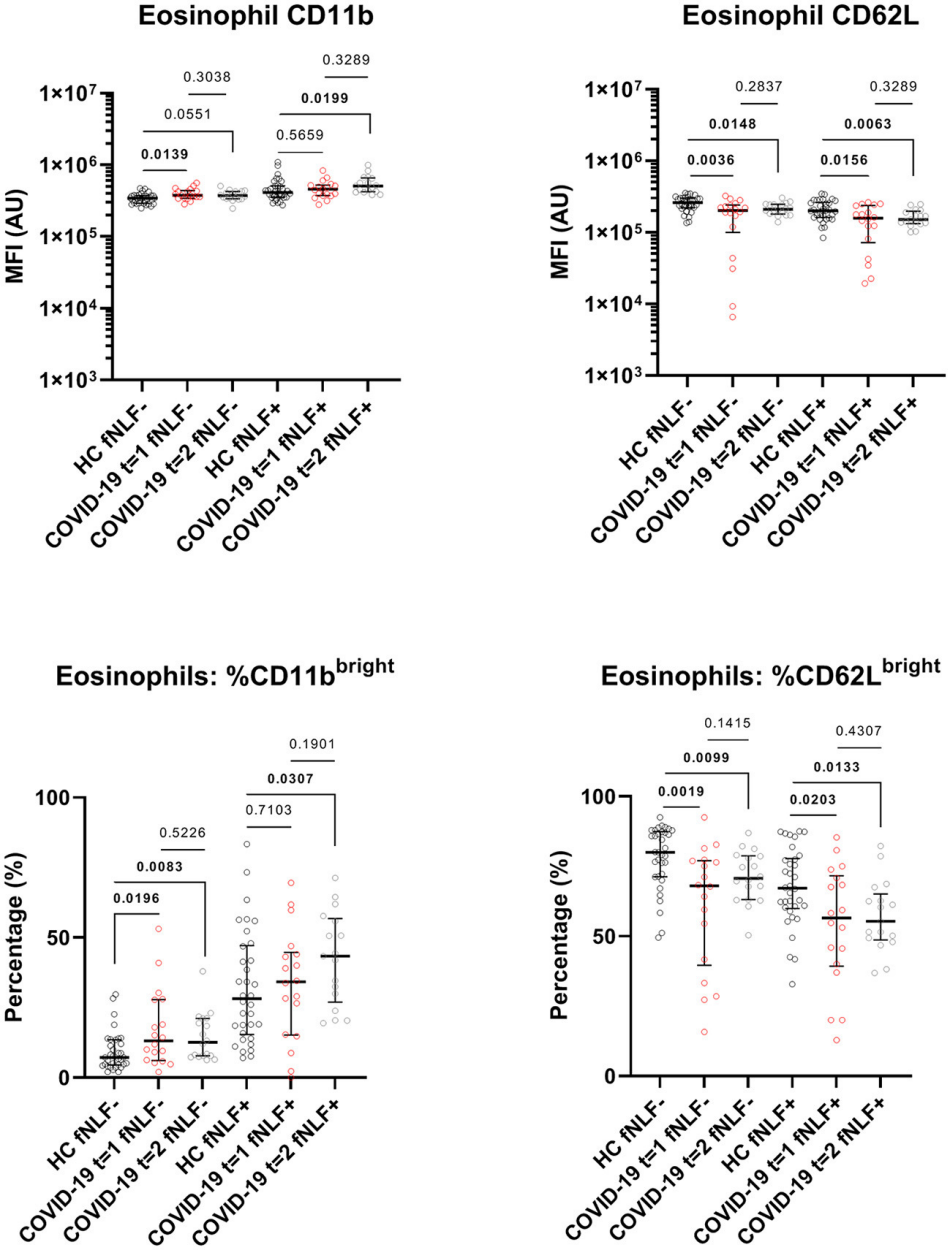
Median fluorescence intensity (MFI) in arbitrary units (AU) for markers on eosinophils during (*t* = 1) and 3 to 6 months after (*t* = 2) active COVID-19 disease in primary care patients. Healthy controls (HC) are displayed as reference. fNLF-samples are measured in the absence of fNLF, whereas fNLF+ samples are measured in the presence of the formylpeptide (10 μM). Statistical significance was tested using the Mann-Whitney U Test for continuous variables and the Wilcoxon matched-pairs signed rank test for paired analyses.

The phenotyping of eosinophils from patients with active COVID-19 disease and eosinophils from healthy controls showed increased CD11b expression [median MFI, 37 x 10^4^ (IQR 34 x 10^4^-44 x 10^4^) vs. 34 x 10^4^ (IQR 29 x 10^4^- 37 x 10^4^), *p* = 0.0139] and a higher percentage of CD11b^bright^ eosinophils in patients with active COVID-19 disease [median percentage, 13.1% (IQR 6.2–27.9%) vs. 7.2 % (IQR 4.6–13.5%), *p* = 0.0196]. This corresponded with the finding of a lowered CD62L expression [median MFI, 20 x 10^4^ (IQR 99 x 10^3^-24 x10^4^) vs. 26 x 10^4^ (IQR 22 x 10^4^-30 x 10^4^), *p* = 0.0036] and a lower percentage of CD62L^bright^ eosinophils in COVID-19 patients with active disease when compared with cells obtained from healthy controls [median percentage, 68.1% (IQR 39.6–77.1%) vs. 80.1% (IQR 71.3–87.4%), *p* = 0.0019, Figure 6].

Also, the responsiveness of eosinophils for fNLF was higher (primed) in patients with active COVID-19 disease. This was illustrated by a more pronounced fNLF down-regulation of CD62L on eosinophils during the active disease phase compared to healthy controls (median MFI, 16 x 10^4^ [IQR 72 x 10^3^-24 x 10^4^) vs. 20 x 10^4^ (IQR 16 x 10^4^- 26 x 10^4^), *p* = 0.0156]. This corresponded with a lower percentage of CD62L^bright^ cells [median percentage, 56.5% (IQR 39.3–71.6%) vs. 67.2% (IQR 60.0–77.8%), *p* = 0.0203, Figure 6] in patients with active COVID-19 disease. Expression of CD11b on stimulated eosinophils as well as the number of CD11b^bright^ eosinophils after fNLF were similar between cells from COVID-19 patients and healthy controls.

During active COVID-19 disease, eosinophil blood counts were reduced and showed baseline activation. The fNLF-stimulated eosinophils of COVID-19 patients with active disease showed signs of increased responsiveness when compared to healthy controls.

#### Eosinophil Surface Markers Are Still Affected 3 to 6 Months After Active COVID-19 Disease

Three to six months after COVID-19 disease unstimulated eosinophils still had a lowered expression of CD62L [median MFI, 21 x 10^4^ (IQR 18 x 10^4^-25 x 10^4^) vs. 26 x 10^4^ (IQR 22 x 10^4^-30 x 10^4^), *p* = 0.0148] and a lower percentage of CD62L^bright^ cells [median percentage, 70.7% (IQR 63.12–78.8%) vs. 80.1% (IQR 71.3–87.4%), *p* = 0.0099, Figure 6] when compared to healthy controls. Accordingly, the percentage of unstimulated CD11b^bright^ eosinophils was increased in COVID-19 patients 3 to 6 months after active disease when compared to healthy controls [median percentage, 12.6% (IQR 7.8–21.2%) vs. 7.2% (IQR 4.6–13.5%), *p* = 0.0083].

Three to six months after COVID-19 disease, the fNLF-stimulated eosinophils had decreased CD62L levels [median MFI, 15 x 10^4^ (IQR 13 x 10^4^-20 x 10^4^) vs. 20 x 10^4^ (IQR 16 x 10^4^-26 x 10^4^), *p* = 0.0063] and a decreased percentage of CD62L^bright^ cells [median percentage, 55.4% (IQR 48.7–65.11%) vs. 67.2% (IQR 60.0–77.8%), *p* = 0.0133] when compared to healthy controls. Stimulated eosinophil CD11b levels [median MFI, 50 x 10^4^ (IQR 42 x 10^4^-66 x 10^4^) vs. 41 x 10^4^ (IQR 35 x 10^4^-51 x 10^4^), *p* = 0.0199] and the percentage of CD11b^bright^ eosinophils were increased [median percentage, 43.4% (IQR 26.9–56.8%) vs. 28.2% (IQR 15.4–47.2%), *p* = 0.0307].

Eosinophils appeared to be more sensitive to activation by fNLF 3 to 6 months after COVID-19 when compared to healthy controls.

#### Eosinophil CD11b and CD62L Expression Did Not Shift in 3 to 6 Months After Active COVID-19 Disease

Eosinophil CD11b and CD62L levels and CD11b^bright^ and CD62L^bright^ percentages were similar during and 3 to 6 months after active COVID-19 disease (Figure 6).

### Granulocyte Surface Markers in Relation to (Prolonged) Disease Symptoms

#### Association of Baseline Expression of CD11b on Neutrophils With Prolonged Duration

**Figure 7:**
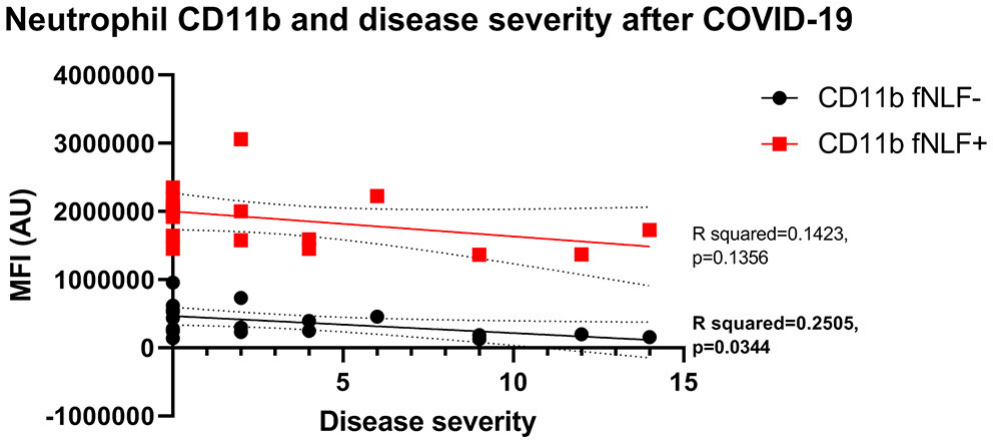
Median fluorescence intensity (MFI) in arbitrary units (AU) in relation to disease severity for neutrophil CD11b 3 to 6 months after active COVID-19 disease. fNLF- samples are measured in the absence of fNLF, whereas fNLF+ samples are measured in the presence of the formylpeptide (10 μM). Trendlines were generated using a simple linear regression analysis.

Both unstimulated and stimulated eosinophil and neutrophil surface markers were not associated with disease severity during active COVID-19 disease. Baseline neutrophil CD11b expression was negatively associated with prolonged symptoms 3 to 6 months after COVID-19 disease (simple linear regression, R squared = 0.2505, *p* = 0.0344, Figure 7). Other neutrophil and eosinophil markers revealed no significant associations.

## Discussion

The aim of this study was to investigate whether the neutrophil and eosinophil compartment were affected in primary care patients with COVID-19 disease. Analysis of 18 primary care patients with COVID-19 disease by fast automated flow cytometry revealed changes in the innate immune system that were characterized by aberrant activation of mature neutrophils and eosinophils. Importantly, 3 to 6 months after active disease there were still indications that the granulocyte compartment was not normalized. Our results suggest that the granulocyte compartment is affected by COVID-19, also in primary care patients outside the hospital.

As described previously, COVID-19 is associated with marked changes in innate immune responses (4–8). These studies, however, mainly included hospitalized, more severely ill patients. The vast majority of COVID-19 patients, however, are managed in primary care. It is, therefore, critical to investigate the pathogenesis of COVID-19 in patients in primary care to unravel the pathogenesis of COVID-19 disease. Eventually this may lead to improved health care as the identification of therapeutic targets will be facilitated with increased knowledge of the disease. Moreover, improved insight into which patients are at risk could facilitate prompt referral and improve prognosis in COVID-19 patients.

### Aberrant Neutrophil Responses in COVID-19 Disease in Primary Care

During active COVID-19 disease in primary care neutrophils showed increased levels of CD11b and decreased levels of CD10 and CD62L. After fNLF activation, CD11b expression further increased and CD62L further decreased to similar levels as found for healthy controls. CD11b and CD62L are well known activation markers for neutrophils (11, 13). This illustrates that neutrophils are activated and responsive in this cohort of primary care patients with COVID-19 disease, pointing at a possible role for neutrophils in COVID-19 pathogenesis in mild disease.

More recently, neutrophil CD10 was also found to be an activation marker (11). Under healthy conditions, a positive correlation was found between the expression of activation markers CD11b and CD10 on *in vitro* activated neutrophils (11). This coincided with down regulation of CD62L. Our data of the healthy controls obtained in this study (healthy individuals participating in the Nijmegen Exercise Study 2021) showed the same characteristics as found in healthy conditions. The baseline decreased CD10 levels on neutrophils in patients with COVID-19 appeared to be in contrast to the normal situation. Furthermore, CD10 was not upregulated to the same extent as found in healthy controls.

This study illustrates that, even though the expression of the classical activation markers CD11b and CD62L is normally regulated, mature neutrophils in COVID-19 patients remain partially refractory to activation by damage associated molecular patterns such as mitochondrial derived formyl-peptides. An explanation could be that COVID-19 directly affected mature neutrophil marker expression and upregulation. This, however, is unlikely, as SARS-CoV-2 enters cells via the angiotensin-converting enzyme 2 receptor and this receptor is not found on neutrophils (14, 15).

CD10 is also a differentiation marker for neutrophils, being upregulated in the latest stage of differentiation (16–18). The lowered expression of CD10 in CD11b^bright^ and CD16^bright^ neutrophils hints toward maturation dissociation characterized by the presence of both young (CD10^dim^) and mature (CD16^bright^/CD11b^bright^) markers on the same cell. This dissociation is not specific for mild disease as similar cells have also been found in moderate to critical disease (4).

Neutrophils contain secretory vesicles and at least three granule subsets (azurophil, specific and gelatinase-containing) (19). It has been suggested that the granular (protein) content is not actively sorted into granules, but that this process is dependent on the proteins that are synthesized in the neutrophil at the time of formation of the different granules (20). Azurophil granules are synthesized in myeloblasts and promyelocytes, specific granules are synthesized in the (meta)myelocyte stages of maturation, gelatinase containing granules are mainly synthesized in band and segmented cells and secretory vesicles are synthesized in segmented neutrophils (21, 22). CD11b, CD16 and CD10 are found in specific granules, gelatinase containing granules and secretory vesicles (19, 23). The distribution of these proteins over these granules and vesicles is heterogeneous: CD11b is well presented in specific and gelatinase containing granules, CD16 is well presented in both gelatinase granules and secretory vesicles, whilst CD10 is predominantly found in secretory vesicles (19). Together, these data support the hypothesis that the lowered CD10 expression in CD16^bright^/CD11b^bright^ neutrophils in patients with COVID-19 is the result of a maturation dissociation in the latest stages of neutrophil development, in which only the secretory vesicles of these young mature neutrophils are not fully synthesized yet.

### Aberrant Eosinophil Responses in Active COVID-19 Disease in Primary Care Patients

The eosinophilic part of the granulocyte blood compartment also showed distinct changes in COVID-19 patients. Lowered eosinophil blood counts are commonly found in COVID-19 patients and the extend of eosinopenia is found to be a marker for severe disease (9, 24–26). This study found, however, that even in a population of mildly ill COVID-19 patients, eosinophil blood counts were lower during the infection than 3 to 6 months after COVID-19, placing this phenomenon in an even wider spectrum of COVID-19 severity.

Even though eosinophils numbers were decreased, more detailed analysis of these eosinophils in primary care patients with COVID-19 revealed increased CD11b levels, whilst having decreased levels of CD62L, indicating baseline activation. Another study also found increased eosinophil CD11b levels in moderately ill, hospitalized COVID-19 patients, but contrarily to our data, found increased CD62L levels in these patients (26). In accordance with our data, lowered CD62L expression on eosinophils with increased CD11b expression was also seen in another study conducted with hospitalized COVID-19 patients (9). This study also found that CD11b^moderate^/CD62L^bright^ eosinophils were unresponsive to fNLF activation. CD62L^bright^ eosinophils have been described as resident, homeostatic eosinophils which are IL-5 independent (27). Combined, these data suggest that the reactive eosinophils disappear from the blood during severe COVID-19 disease, leaving behind the resident eosinophils, whilst this does not seem to happen in primary care patients with milder symptoms.

Activation with fNLF showed increased responsiveness of eosinophils as CD62L was downregulated to a bigger extent when compared to healthy controls. Apart from these eosinophils still being responsive, this also hints toward a higher sensitivity or priming for (*ex vivo*) activation. A study on eosinophilic responsiveness to fNLF in hospitalized COVID-19 patients distinguished two subgroups of eosinophils: responsive and unresponsive cells (9). The authors found that eosinophilic reactivity increased with disease resolution. Accordingly, in our study primary care COVID-19 patients with mild symptoms showed to have responsive eosinophils. Furthermore, our data suggest that these responsive eosinophils are already baseline activated (high CD11b expression, lowered CD62L expression) during the COVID-19 infection, supporting the concept that eosinophils play a role in the pathogenesis of (mild) COVID-19. Our data are in agreement with the results of another study that describes activation of the IFN-γ-eosinophil axis in COVID-19 that precedes lung hyper-inflammation (28). It is tempting to speculate that eosinophils directly affect the course of COVID-19 as eosinophils have also been described to be involved in killing bacteria and (respiratory) viruses and act by producing antiviral molecules, but also by serving as antigen-presenting cells (29–31).

### Aberrant Granulocyte Responses Months After Active COVID-19 Disease

Even though some activation markers normalized months after active COVID-19, the neutrophil compartment was still activated and did not fully normalize within months after the infection. Baseline neutrophil CD62L expression was still decreased and similar to expression during active disease.

Circulatory CD62L^dim^/CD16^bright^ “hypersegmented” neutrophils have been described in the context of trauma, experimental endotoxemia and exercise (32–34). “Hypersegmented” neutrophils are thought to be a truly separate subset that has poor bacterial killing capacities and inhibits T-cell proliferation (35–37). A less cytotoxic and more regulatory function for these neutrophils might be beneficial in the period after active disease.

fNLF-stimulation of neutrophils post COVID-19 resulted in decreased levels of CD10 compared to control levels, indicating the persistent incapacity of these neutrophils to fully respond to activation by fNLF. Both un- and stimulated neutrophil CD10 levels increased after COVID-19 when compared to during active disease, but had not reached the levels such as found in normal controls. This suggests that young-mature neutrophils are still present up to 6 months after active COVID-19 disease, but to a lesser extent than during active disease. This indicates that the neutrophil compartment is long-term affected by COVID-19, even in patients with lower disease severity.

CD16^bright^/CD10^dim^ neutrophils are thought to have a different function than CD16^bright^/CD10^bright^ neutrophils. Whilst “normal” neutrophils phagocyte debris, remove dead tissue and make channels for angiogenesis, CD10^dim^ neutrophils promote T-cell survival and stimulate IFN-γ production by T-cells (38, 39). IFN-γ production is critical for innate and adaptive viral immunity, which supports the concept that the neutrophil compartment is more involved in immune regulation rather than classical cytotoxicity.

The eosinophil data illustrate that even 3 to 6 months after COVID-19, activation and hyper-responsiveness/priming for formylpeptides is present in the eosinophilic compartment. It seems counterintuitive that blood eosinophils from milder COVID-19 patients exhibited a more activated phenotype than patients with more severe disease. However, several lines of evidence support the hypothesis that activated eosinophils leave the blood during active disease, leaving behind low numbers of less active cells in the peripheral blood (40, 41).

### Putative Correlation Between Persistent Complaints and Aberrancies in the Innate Immune System

The long term impact of COVID-19 on neutrophils and eosinophils has not been described before. This study revealed that mild COVID-19 in primary care patients was associated with long term effects on eosinophils and neutrophils. Similar findings were obtained with NKT-cells. In a population of COVID-19 patients in which 43% were hospitalized, the long term impact of COVID-19 was associated with decreased NKT(-like)-cell frequencies 2 months post-COVID-19 (42). Combined, this indicates that the innate immune system is still affected by COVID-19 up to months after the initial infection.

Patients in this study reported symptoms in varying degrees during and 3 to 6 months after COVID-19. Baseline neutrophil CD11b expression 3 to 6 months after COVID-19 was negatively associated with prolonged symptoms, again suggesting that active neutrophils, just like eosinophils, home to the tissues in active disease. This might, therefore, point at the presence of residual disease months after the infection. It is possible that COVID-19 causes long-term tissue damage and production of damage associated molecular patterns (DAMPs), to which neutrophils react, because of their role in tissue repair and regeneration (43). The persistence of some of these cells in the blood might be a signal that some COVID-19 dependent mechanisms could still be found in the body to which these young-mature neutrophils react.

The baseline activation of eosinophils and their hyper-responsiveness toward fNLF after COVID-19 indicate that also eosinophils respond to similar triggers as found for neutrophils. Apart from anti-bacterial and anti-viral capabilities, eosinophils can also exhibit immune-regulatory functions (29–31, 44). Direct anti-SARS-CoV-2 activity by eosinophils is possible as antiviral activity by human eosinophils has recently been shown. However, direct killing of SARS-CoV2 by eosinophils has not yet been observed in humans (44). On the other hand, eosinophil-mediated inflammation has been associated with critical COVID-19 disease (45). It is, therefore, more likely that the immune-regulatory functions of eosinophils are involved in the regulation of the malfunction of the immune response in COVID-19 rather than direct anti-viral activity.

## Strengths and Limitations

A strength of our study was that, to our knowledge, this is the first study to investigate the immunological implications of COVID-19 on the granulocyte blood compartment during and after relatively mild disease in primary care patients. The paired analyses of the same patients during and after COVID-19 allows to draw conclusions regarding the changes in the granulocyte compartment during the course of mild COVID-19 disease in primary care.

However, some limitations need to be considered. Firstly, the generalizability of the results of this study is limited due to its small cohort size. Secondly, the need of a healthy control cohort due to the lack of pre-COVID-19 disease measurements of the COVID-19 patients could be seen as a limitation.

## Implications for Clinical Practice

At this stage the implications for clinical practice are premature. Yet, a more extensive study will focus on the longer term effects of COVID-19 on granulocyte surface markers. Whether these markers still show altered expression 2 years after the initial infection and whether a clinical marker for patients at risk for hospitalization will be found will be investigated.

## Conclusion

This small cohort study revealed that the granulocyte blood compartment is affected during and 3 to 6 months after active COVID-19 disease in primary care patients. During and 3 to 6 months after COVID-19 the neutrophil compartment was characterized by activation patterns and a counterintuitive lowered CD10 expression, suggesting the presence of young-mature neutrophils which could act as pro-inflammatory cells. The hyper-responsive eosinophilic compartment hints toward a more immune-regulatory function of these cells in primary care patients with COVID-19 disease. As the granulocyte compartment was affected also in mild COVID-19 disease in primary care, its role in the pathogenesis should be further investigated.

## Data Availability Statement

The raw data supporting the conclusions of this article will be made available by the authors, without undue reservation.

## Ethics Statement

The studies involving human participants were reviewed and approved by Medical Ethics Review Committee Utrecht. The patients/participants provided their written informed consent to participate in this study.

## Author Contributions

Conceptualization: LK, RV, DZ, FR, CB, and MH. Methodology - COVID-19 patients and analysis: BJ and KS. Methodology - healthy controls: BJ, TCP, CB, and MH. Writing – original draft preparation: BJ, LK, and KS. Writing – review and editing: BJ, LK, KS, MH, CB, MR, TNP, RV, DZ, and FR. Visualization: BJ. Supervision: LK, MH, CB, RV, DZ, and FR. All authors have read and agreed to the published version of the manuscript. All authors contributed to the article and approved the submitted version.

## Funding

This study was partially funded by the foundation Hartstichting and foundation Stoffels-Hornstra, both residing in the Netherlands and ZonMw- the Netherlands Organization for Health Research and Development-Promotieonderzoek aios Huisartsgeneeskunde 2021 (grant 08391052110003).

## Conflict of Interest

The AQUIOS CL^®^ “Load & Go” flow cytometer is provided by the company Beckman Coulter Life Sciences, Miami, FL, USA. The authors declare that the research was conducted in the absence of any commercial or financial relationships that could be construed as a potential conflict of interest.

## Publisher's Note

All claims expressed in this article are solely those of the authors and do not necessarily represent those of their affiliated organizations, or those of the publisher, the editors and the reviewers. Any product that may be evaluated in this article, or claim that may be made by its manufacturer, is not guaranteed or endorsed by the publisher.

## Supplementary Material

Supplementary Figure 1 Strategy for identifying the purple FlowSOM meta-cluster in the primary panel as eosinophils. Combining the clustering data from both the granulocyte and eosinophil panel with(out) CD193, the purple meta-cluster contains CD11b^high^, CD16^low^ and CD193^high^ cells with a high sideward scatter profile. The purple meta-cluster was therefore identified as eosinophils. The red metacluster also contains CD193^high^ cells. The typical diagonal pattern of the cells in the red metacluster for several markers with a sideward scatter profile that is beyond the axis limit makes it most likely that it consists of dead, sticky cells.

Supplementary Figure 2 Gating strategy for defining CD11b^bright^ and CD62L^bright^ eosinophils. The gates were set at predefined Median Fluorescent Intensities (MFI): 547873 for CD11b and 136968 for CD62L. Activated refers to samples measured in the presence of the formylpeptide fNLF (10 μM).
