# Supplementary material for: Neutrophil and Eosinophil Responses Remain Abnormal for Several Months in Primary Care Patients With COVID-19 Disease
Source: Front Allergy. 2022 Jul 27;3:942699. doi: 10.3389/falgy.2022.942699 (PMC9365032; doi:10.3389/falgy.2022.942699)
Supplement: Supplementary file 1 [file Table_1.DOCX]

**Patient reported outcome-COVID-19 questionnaire**

#
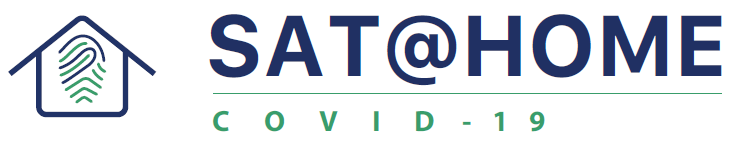
 COVIDSat@Home

During and after COVID-19 disease episode

Answer de questions per symptom twice. The questions concern the symptoms you:

1. Experienced during your active COVID-19 disease episode between January 1th and June 1th 2021 and;
2. Are (still) experiencing at the moment (after 3-6 months)

| Symptom | Not at all | A little | Moderately | Quite a bit | Extremely | How long before symptoms subsided (weeks) | How long before symptoms subsided (days) |
| --- | --- | --- | --- | --- | --- | --- | --- |
| Coughing | 1 | 2 | 3 | 4 | 5 | If applicable | If applicable |
| Chest pains | 1 | 2 | 3 | 4 | 5 | If applicable | If applicable |
| Shortness of breath | 1 | 2 | 3 | 4 | 5 | If applicable | If applicable |
| Coughing up phlegm/sputum | 1 | 2 | 3 | 4 | 5 | If applicable | If applicable |
| Coughing up blood | 1 | 2 | 3 | 4 | 5 | If applicable | If applicable |
| Sweating | 1 | 2 | 3 | 4 | 5 | If applicable | If applicable |
| Chills | 1 | 2 | 3 | 4 | 5 | If applicable | If applicable |
| Headache | 1 | 2 | 3 | 4 | 5 | If applicable | If applicable |
| Nausea | 1 | 2 | 3 | 4 | 5 | If applicable | If applicable |
| Vomiting | 1 | 2 | 3 | 4 | 5 | If applicable | If applicable |
| Diarrhea | 1 | 2 | 3 | 4 | 5 | If applicable | If applicable |
| Stomach pain | 1 | 2 | 3 | 4 | 5 | If applicable | If applicable |
| Muscle pain | 1 | 2 | 3 | 4 | 5 | If applicable | If applicable |
| Lack of appetite | 1 | 2 | 3 | 4 | 5 | If applicable | If applicable |
| Trouble concentrating | 1 | 2 | 3 | 4 | 5 | If applicable | If applicable |
| Trouble thinking | 1 | 2 | 3 | 4 | 5 | If applicable | If applicable |
| Trouble sleeping | 1 | 2 | 3 | 4 | 5 | If applicable | If applicable |
| Fatigue | 1 | 2 | 3 | 4 | 5 | If applicable | If applicable |
| Fever | 1 | 2 | 3 | 4 | 5 | If applicable | If applicable |
| Diminished smell and/or taste | 1 | 2 | 3 | 4 | 5 | If applicable | If applicable |
| Other symptoms | 1 | 2 | 3 | 4 | 5 | If applicable | If applicable |
