# Supplementary figures and images for: Neutrophil and Eosinophil Responses Remain Abnormal for Several Months in Primary Care Patients With COVID-19 Disease
Source: Front Allergy. 2022 Jul 27;3:942699. doi: 10.3389/falgy.2022.942699 (PMC9365032; doi:10.3389/falgy.2022.942699)

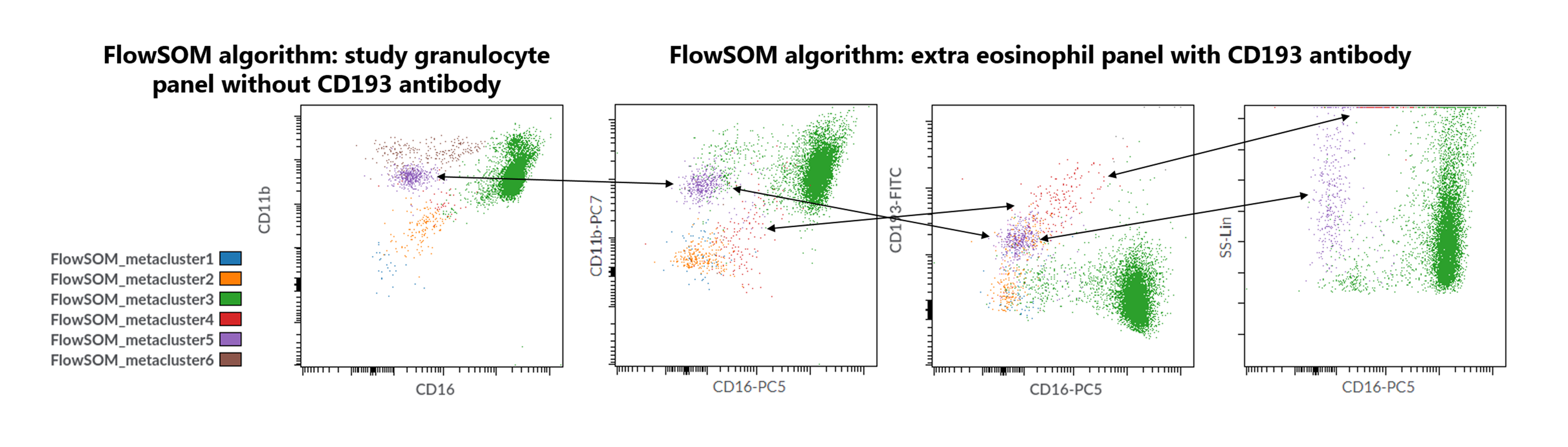

Supplement: Supplementary Figure 1 — Strategy for identifying the purple FlowSOM meta-cluster in the primary panel as eosinophils. Combining the clustering data from both the granulocyte and eosinophil panel with(out) CD193, the purple meta-cluster contains CD11bhigh, CD16low and CD193high cells with a high sideward scatter profile. The purple meta-cluster was therefore identified as eosinophils. The red metacluster also contains CD193high cells. The typical diagonal pattern of the cells in the red metacluster for several markers with a sideward scatter profile that is beyond the axis limit makes it most likely that it consists of dead, sticky cells. [file Image_1.TIF]

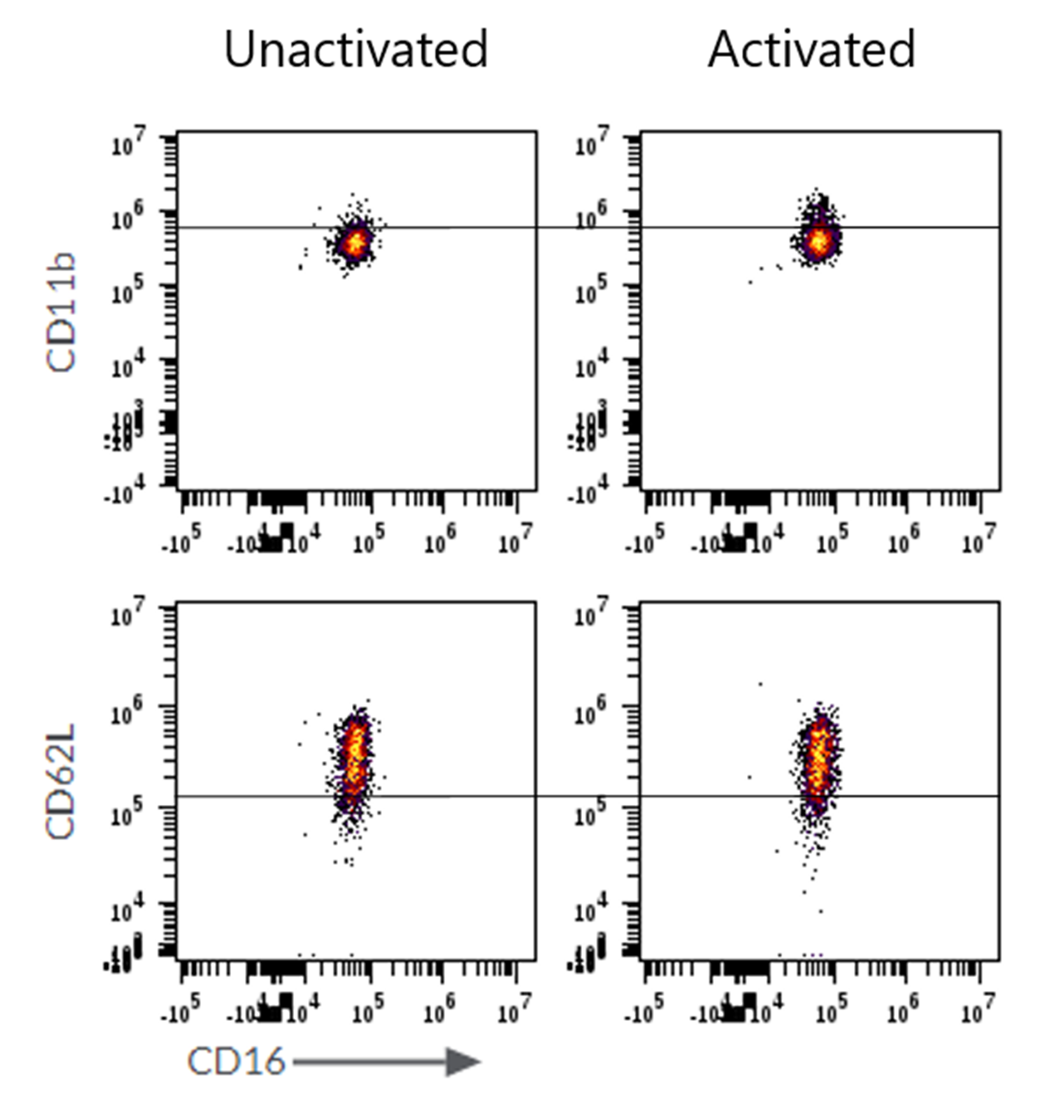

Supplement: Supplementary Figure 2 — Gating strategy for defining CD11bbright and CD62Lbright eosinophils. The gates were set at predefined Median Fluorescent Intensities (MFI): 547873 for CD11b and 136968 for CD62L. Activated refers to samples measured in the presence of the formylpeptide fNLF (10 μM). [file Image_2.TIF]
